# Carbon Dioxide Uptake Estimation for Spanish Cement-Based Materials

**DOI:** 10.3390/ma17020326

**Published:** 2024-01-09

**Authors:** Natalia Sanjuán, Pedro Mora, Miguel Ángel Sanjuán, Aniceto Zaragoza

**Affiliations:** 1 Civil Engineering School, Technical University of Madrid (UPM), C/Professor Aranguren, 3, University City, 28040 Madrid, Spain; natalia.sanjuan@alumnos.upm.es; 2 Department of Geological and Mines Engineering, Mine and Energy Engineering School, Technical University of Madrid (UPM), C/Ríos Rosas, 21, 28003 Madrid, Spain; pedro.mora@upm.es; 3 Spanish Institute of Cement and Its Applications (IECA), C/José Abascal, 53, 28003 Madrid, Spain; 4 Oficemen, C/José Abascal, 53, 28003 Madrid, Spain; azaragoza@oficemen.com

**Keywords:** carbon dioxide uptake, building materials, cement, Roadmap 2050, policy

## Abstract

The Intergovernmental Panel on Climate Change (IPCC), which is the United Nations body for assessing the science related to climate change, has recently recognized the natural carbonation process as a way of carbon offsetting with mortar and concrete. Accordingly, this activity could be recognized as a carbon removal process for which certification should be granted. The aim of the certification of carbon removal is to promote the development of adequate and efficient new carbon removal processes. Therefore, the main objective of this study is to provide reliable results on carbon dioxide uptake by cement-based materials in Spain. Yearly, greenhouse gas emissions are reported to the United Nations Framework Convention on Climate Change (UNFCCC) by each country, and the natural carbonation should be added up to the carbon accounting. Therefore, natural carbonation should be included in the IPCC Guidelines for National Greenhouse Gas Inventories, and such accounting information should be made available promptly to the national regulatory authorities. This paper provides the results of carbon dioxide uptake by Spanish cement-based materials from 1990 to 2020 by using an easy method of estimating the net carbon dioxide emissions (simplified method) considering the carbon dioxide released by the calcination during clinker production (process emissions). The outcome of this study reveals that there was 93,556,000 tons of carbon dioxide uptake by the mortar and concrete manufactured in Spain from 1990 to 2020.

## 1. Introduction

Within the wider context set by the European Union (EU) Strategic Agenda to become climate neutral by 2050, the EU has committed itself to reduce its net greenhouse gas emissions (GHG) by at least 55% by 2030, compared to 1990 levels [1,2]. The European Climate Law [3] requires Member States to balance greenhouse gas emissions and removals within the European Union at the latest by 2050, i.e., lowering emissions to net zero by that date, and to reach negative emissions thereafter. Nevertheless, Europe only produces 5% of the global cement output, whereas China is the largest producer in the world (54% in 2020) [4].

China continues to honor all its commitments made under the Paris agreement to cut its carbon dioxide emissions per unit of gross domestic product (GDP) by 60–65% from 2005 level by 2030 [5]. Recently, China has committed itself to the goal of achieving carbon neutrality by 2060 according to its Climate Green Deal published in September 2020 [5]. Currently, China emits more than 10 Gt of carbon dioxide yearly, i.e., one third of the worldwide carbon dioxide emissions [6].

On the other hand, China’s Cement Industry (CCI) emissions represent over 15% of Chinese carbon dioxide emissions [7], and about 60% of global cement sector emissions [8] since Chinese Portland cement production represents 52% of the global production (4650 million tons in 2016) [8]. It is noticeable that China’s cement production increased by seven fold between 1990 and 2019 [9]. Consequently, carbon dioxide emissions also increased around 300%, i.e., from 400 Mt (1990) to 1300 Mt (2020) [10,11]. From this we can conclude that the Chinese Cement Industry plays a key role to achieve the carbon neutrality by 2060 in China. According to Dinga and Wen [12], the achievement of carbon neutrality by China’s Cement Industry by 2060 is ambitious but possible under advanced emission abatement measures. Wei et al. [5] suggested that China’s cement carbon dioxide emission factors will be around 390–407 kg CO_2_/t of cement.

GHG emissions must be sharply reduced across all sectors and, in accordance with The European Union guidelines [2] among those of other relevant international bodies, all industrial value chains must work on reducing their own carbon footprints but also accelerate the transition by developing new technology and new business models. In particular, the built environment is a major source of carbon dioxide emissions. Strategically, the building sector together with policymakers must be given the tools to properly reduce resource use, energy, and carbon emissions, in order to minimize the social, environmental, and economic impacts of climate change. Accordingly, it is necessary to set up an adequate policy framework to enhance new technologies and methodologies that would finally allow the cement industry to achieve a huge reduction in carbon dioxide emissions [13]. In particular, the worldwide cement sector can offer some carbon removal down the cement and concrete value chain:

- In integrated Portland cement plants, i.e., factories with clinker production: **Carbon Capture, Utilization, and Storage (CCUS)** and **biomass waste** use as a non-recyclable-waste-derived fuel. Currently, biomass use in Spain accounts for 33% of non-recyclable-waste-derived fuels, whereas in Europe (CEMBUREAU) it accounts for 17%. The global non-recyclable-waste-derived fuels percentage in Spain is 37%, while in Europe (CEMBUREAU) it is 52%. Furthermore, the Spanish carbon neutrality roadmap aims to achieve 70% alternative fuel usage by 2050, of which almost half (about 40%) should be biomass-waste-derived fuels. In Europe (CEMBUREAU), the use of alternative fuels is expected to reach about 90%, with more than 50% being biomass waste.
- In mortar and concrete through their service life: **natural carbonation.**
- In concrete production (ready-mix concrete or precast concrete): **controlled accelerated concrete carbonation** permanently stores carbon and increases the concrete compressive strength. This notwithstanding, this process enables the use of cements with a lower content of clinker in the concrete, which leads to a further reduction of carbon dioxide in the built environment. It is well-known that carbonation results in increased compressive strength for CEM I concrete. By contrast, concrete manufactured with blended cements present a lower compressive strength than CEM I concrete.

Consequently, these three activities should be adopted by the new European Union (EU) carbon removal framework [14,15], which are in line with the Carbon Neutrality Roadmap of the Spanish cement industry published in December 2020 [16,17] and that of the European cement industry published in May 2020 [18]. Several researchers [12,17] support the proposal to recognize such activities as carbon removal processes for which certification should be granted. The proposal for the certification of carbon removal has the aim of promoting the development of new carbon removal processes in line with the European Climate Law [3] and European Green Deal objectives [19]. In particular, capture and storage of biogenic carbon dioxide from Portland cement plants could qualify as carbon removal according to Art. 1(2) of the EU carbon removal framework proposal [14], whereas the use of biogenic carbon dioxide to enhance the concrete carbonation should be qualified as carbon removal according to Art. 2(i) of the EU carbon removal framework proposal, i.e., carbon storage in mortar and concrete by carbonation represents a carbon removal procedure that stores atmospheric carbon dioxide in long-lasting storage.

Furthermore, the “Proposal for a regulation of the European Parliament and of the Council establishing a Union certification framework for carbon removals” has been recently amended by the European Parliament by adding “… carbon farming and carbon storage in products” [20]. This fact indicates the significance of the carbon storage in products such as concrete and aims to expedite the path towards reaching or over-achieving the target of Climate Neutrality by 2025 and accomplishing the objective of sequestering carbon dioxide. To sum up, concrete carbonation encompasses two key types of carbon removal activities recognized by the voluntary EU-wide framework: permanent storage and carbon storage in products [14]. In addition, certificates earned in the EU carbon removal context that stem from the Emission Trading System (ETS) activities should be accountable in the European Emission Trading System (EU ETS) framework.

Meanwhile, The Intergovernmental Panel on Climate Change (IPCC) has recognized the natural carbonation process as a way of carbon offsetting with mortar and concrete in the Working Group I contribution to the Sixth Assessment Report (AR6) [21].

In addition, the Working Group III contribution to the Sixth Assessment Report (AR6) [22] underlines that the urban infrastructures containing cement-based materials uptake carbon dioxide through the process of carbonation. The concept of buildings as carbon sinks rises from the idea that wood stores carbon [22], but not permanently. By contrast, concrete has a permanent carbon storage capacity. However, the natural carbonation process is not yet included in the IPCC Guidelines for National Greenhouse Gas Inventories as a form of carbon removal [23,24], but it is nevertheless going in the right direction according to The European Cement Association (CEMBUREAU), which includes carbonation as the fifth step in its Carbon Neutrality Roadmap [18].

### 1.1. Whole-Life Carbon Emissions of Buildings and Civil Constructions

Carbon neutrality by 2050 requires the consideration of both embodied and operational carbon emissions in a “whole life carbon”, covering all carbon dioxide emissions derived from the material production phase, construction phase, and usage phase over its service life, including the end-of-life phase, i.e., demolition and disposal [25].

**Figure 1:**
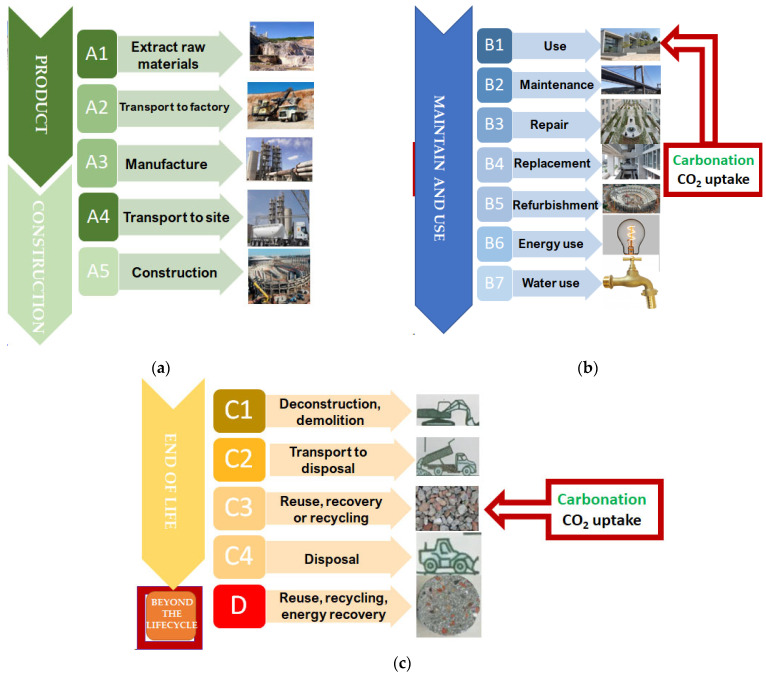
Life-cycle modules according to the European standard EN 15978. Building assessment information: (**a**) production (A1–A3) and construction processes (A4–A5); (**b**) ‘in-use’ stages (B1–B7) (**c**) ‘end-of-life’ stages (C1–C4) and supplementary information (D).

The European standard EN 15978 affords a framework for addressing issues relating to the environmental performance of buildings during their life cycle (Figure 1) and provides a methodology to perform a life-cycle assessment (LCA).

Figure 1 shows the life-cycle modules defined in the European standard EN 15978 [25]. Life-cycle assessment (LCA) applied to cement-based materials, such as mortar and concrete, should consider the carbon dioxide uptake since LCA is the most common systematic procedure of collecting and assessing all the inputs, outputs, and environmental impacts of cement-based materials over its lifetime. Accordingly, LCA is an effective way to measure embodied carbon, but also can include carbon dioxide uptake. According to EN 15978 [25], embodied carbon refers to the GHG emissions associated with the material production (A1–A3) and construction processes (A4–A5) throughout the whole service life of a building construction or civil engineering work. Furthermore, it considers the ‘in-use’ (B1–B5) and ‘end-of-life’ (C1–C4) stages and the associated transportation. By contrast, operational carbon refers to the GHG emissions related to the energy consumed during the use phase, i.e., heating, cooling, lighting, cooking, and so on (B6).

Figure 1 shows all the EN 15978 modules, including the upfront carbon (embodied), which is the carbon dioxide emitted during the stages of material production and building or civil construction before it begins being used (modules A1–3 and A4–5, respectively, in Figure 1).

These stages are considered in the cement 2050 Carbon Neutrality Roadmaps published elsewhere [17,18]. On the other hand, Module D is optional and provides additional information about some potential environmental benefits, such as carbonation of cement-based materials. Natural carbonation is produced during the use (B1) and re-use, recovery, or recycling (C3) stages as shown in Figure 1. Both ways of carbon dioxide uptake are assessed in the present study.

### 1.2. Carbon Dioxide Uptake by Cement-Based Materials

Carbonation is a natural physico-chemical process which occurs during the service life and at the end of use of mortars and concretes. Accordingly, a percentage of carbon dioxide emitted during Portland cement clinker production is absorbed into both materials.

Addressing the current gap between available mitigation techniques for GHG emissions associated with the Portland cement production and the duty to strive for a carbon- and climate-neutral industry represents a relevant challenge. Specific solutions focused on narrowing the gap are necessary. Stefaniuk et al. [26] consider natural carbonation and direct anthropogenic carbon dioxide sequestration and storage in concrete through carbon mineralization forming calcium carbonates such as vaterite, ikaite, and calcite as two potential options. In addition, they remark that the mentioned calcium carbonates can serve as nucleation sites for the generation of composites formed of C-S-H gel and CaCO_3_ [26].

Several studies found in the literature are related to the potential for mineralizing carbon dioxide into concrete through carbonation curing of cement-based materials [27,28,29].

Li et al. [28] found that the compressive strength of carbonation-cured concrete was more than 10% higher than that of moisture-cured concrete at the same age. In addition, this type of concrete was less permeable to chlorides and exhibited better abrasion resistance due to the narrower pore of the carbonation-cured concrete than the plain concrete. By contrast, Zhang et al. [27] believe that carbonation curing is still immature from both technical and economic perspectives. Therefore, the current knowledge about this technique is not sufficient to guarantee a widespread use of carbonation-cured concrete.

With regard to the natural carbonation, the European standard EN 16757:2022 [30], which complements the product category rules (PCR) as given in EN 15804 [31], includes an Annex G for natural carbonation accounting guidelines.

Zhang et al. [27] state in their review on carbonation curing of cement-based materials that carbonation is a natural physico-chemical process whereby carbon dioxide present in the air enters the mortar and concrete capillary pores and reacts with calcium hydroxide, which is present in the pore solution, and with some hydration products [27]. Therefore, EN 16757 affords additional rules for Environmental Product Declarations (EPD) for concrete and concrete elements. In this regard, it should be noted that the impacts generated during the use and end-of-life stages can include concrete carbonation [30], as shown in Figure 1.

The climate impact resulting from the construction materials may be inferred from their global warming potential (GWP), which is a metric for assessing embodied carbon. This climate impact is listed in the environmental product declaration (EPD) along with other impacts.

According to EN 16757:2022 [30], concrete carbonation can be included in the stages of use (Module B1) and end-of-life (C3 “Waste processing” and C4 “Disposal”). Furthermore, precast concrete or ready-mix concrete can be subjected to accelerated carbonation during the production stage by means of CO_2_ curing. Hence, carbon dioxide uptake associated with carbonation may be reported at each of these life-cycle stages following the calculation method provided in Annex G (EN 16757:2022).

It is well-known that the content of bound carbon dioxide changes depending on the type of concrete (dosage, curing conditions, and so on), the environmental conditions at the place of use, and the end-of-life scenario. Thus, Annex G (EN 16757:2022) [30] applies such parameters as they are considered appropriate to the carbon dioxide uptake calculations.

In compliance with the Sixth Assessment Report (AR6) of The Intergovernmental Panel on Climate Change (IPCC), this study covers the relevant questions related to the implementation of the Tier 1 methodology (simplified procedure) for natural carbonation estimation, which can be considered as a way of carbon offsetting.

Accordingly, this paper deals with the analysis of the clinker production, export, and import data to estimate the uptake of carbon dioxide by Spanish cement-based materials made with such clinkers from 1990 to 2020.

## 2. Materials and Methods

### 2.1. Data Collection

Considering that a data collection method is reliable and valid to the extent that it produces the same results repeatedly, the following procedure has been applied. Accordingly, the data collection method employed was based on the following two data sources:

1. Annual compilations and collations of data according to official statistics from the Spanish cement sector (Oficemen), beginning in 1990.
2. Following a prior request by the authors, in some cases, manufacturers collect and send us data.

The authors then carried out an analysis (including, if need be, contact with some manufacturers where the data provided by cement producers differed significantly from those gathered directly by the authors through Eurostat or other international statistics) and an appraisal for the final report.

Consequently, the data used for the carbon dioxide uptake estimation can be considered robust data since 1990, as they correspond to the verified statistics of the Spanish cement sector (Oficemen).

### 2.2. Carbon Dioxide Uptake by Natural Carbonation

#### 2.2.1. Carbonation in the Standardization and Policy Context

Some recent international publications have suggested that, as a first approximation, about 23% of the annual calcination emissions, i.e., carbon dioxide emitted from the Portland cement clinker production, from Portland cements that have been consumed throughout the year will be absorbed by mortars and concretes used in buildings and civil structures [9,32,33] by the well-known physico-chemical process of carbonation [34]. The carbonation process is well-established, and the estimation of the carbon dioxide uptake is already included in the European standard EN 16757 (Annex G) [30]. Furthermore, the carbonation process has been recognized by the Working Group I of the Intergovernmental Panel on Climate Change (IPCC) in its contribution to the Sixth Assessment Report (AR6) [21]. In doing so, the carbon dioxide absorption by mortars and concretes converts the built environment into an outright carbon sink.

#### 2.2.2. Carbonation Modeling

Although many kinetic models have been developed for depicting carbonation [35,36,37,38,39,40], in most of them carbonation proceeds by diffusion according to Fick’s second law of diffusion. For instance, Equation (1) is a solution to Fick’s second law considering moving boundaries [41], where it is assumed that the chemical reaction between carbon dioxide, calcium phases (Ca^2+^), and water (H_2_O) generates an immobile reaction product, calcium carbonate (CaCO_3_) [41]. For mortar and concrete, C_2_ = 0 and C_x_ <<< C_1_.

(1) CX−C1C2−CX=Πxt2Dexpx2t4Derfxt2D≈C1CX,

In this equation, the diffusion coefficient of the carbon dioxide is D (m^2^/s), whereas C_x_ is the CO_2_ concentration at discontinuity (kmol/m^3^), C_1_ is the CO_2_ concentration in surroundings (kmol/m^3^) and C_2_ is the CO_2_ concentration in the Portland-cement-based material (kmol/m^3^). Therefore, the carbonation rate must be determined for each type of concrete.

However, the square root of the time model shown in Equation (2) can accurately predict the cement-based material carbonation rate [36]. Accordingly, this is the most utilized model [42,43], which relates the carbonation depth to the carbonation coefficient, B.

The equation used in this method is the general solution of Fick’s second law of diffusion (Equation (2)), which has been employed extensively in the concrete field. In this model, the influence of complex factors is depicted by a single parameter, B, which is simple in form and easy for calculation. The square root model has been assessed by many studies, concluding that the carbonation depth reflects a linear relationship with the square root of time with correlation coefficients above 97% [44]. This method has been applied to field studies with good results [45].

(2) $$x=B\sqrt{t}$$

In this equation, the carbonation coefficient is B (mm/yr^0.5^), while x (mm) is the carbonation depth and t (year) the natural carbonation exposure time.

The square root of the time function always increases throughout its entire domain (0, ∞), i.e., as x increases, the value of f(x) also increases. However, this function grows faster during the first 50 years of carbonation exposure. As is well-known, a function’s growth rate is a measure of how much a function changes. Within this context, the growth rate of the square root function for carbonation, which is equivalent to its rate of change, slows down from previous trend. Therefore, the estimation is limited to the first 50 years for the cement-based materials carbonation performed in the present study.

#### 2.2.3. Factors Affecting Carbonation

**Figure 2:**
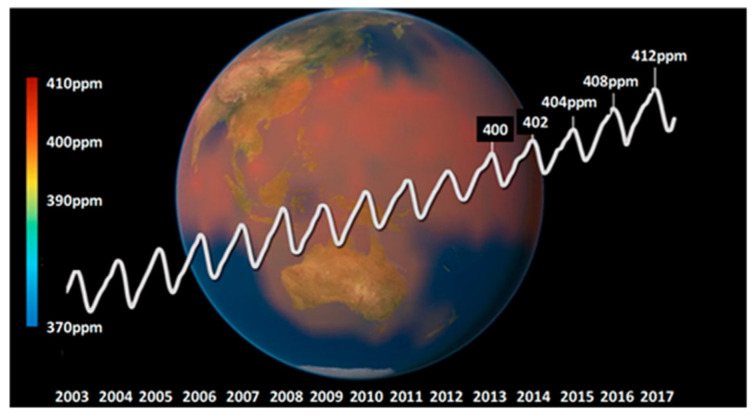
World carbon cycle: concentrations of carbon dioxide in the atmosphere.

Several factors dictate the carbonation level of cement-based materials. A prime parameter of the carbonation phenomenon, in which carbon dioxide in the atmosphere propagates into concrete to react with calcium hydroxide and other compounds to form calcium carbonate, is the carbonation rate. The carbonation coefficient, B (mm/yr^0.5^), depends on several factors such as the air’s relative humidity, the cement type, or the carbon dioxide content in the environment, which varies from 0.03% in rural areas to 0.4% in urban or industrial areas. Furthermore, carbon dioxide concentration has risen substantially in the past one hundred years and especially in the past five decades due to human activities (Figure 2) [8].

The degree of carbonation (DoC) of the concrete is the content of actual carbonated cementitious material compared with the maximum amount that may have been carbonated under optimal conditions. Bui et al. [46] reviewed the applicability of different methods for carbonation degree measurement in concrete. The carbonation degree is normally determined by the level of inward diffusion of carbon dioxide from the concrete surface to the inner zone [47]. On the other hand, to calculate the maximum amount of carbon dioxide that a cement can absorb, it is considered that all the calcium oxide present in the portlandite, Ca(OH)_2_, and the aluminates is carbonated. However, only 59% of the calcium oxide present in the C-S-H gel carbonates. Accordingly, it must be taken into account that only 76% of the CaO present in Portland cement clinker is likely to be carbonated [8].

### 2.3. Carbon Dioxide Uptake Estimation and Hypothesis

The hypotheses set out in the present study for the estimation of carbon dioxide uptake by natural carbonation of cement-based materials produced in Spain from 1990 to 2022 are as follows:

- The data used in the CO_2_ uptake estimation are considered “robust data” since 1990, as they correspond to the verified statistics of the Spanish cement sector. Accordingly, they are “virtually certain” according to the IPCC likelihood scale [48].
- Concrete and mortar to be carbonated have been produced and used in Spain in civil works and building in the years preceding the year concerned.
- The highest growth of the square root of time function is produced during the first years. Therefore, the carbon dioxide uptake estimation has been limited to the first 50 years in this study, considering an average carbonation rate of 3 mm/year^0.5^.
- It has been considered that 23% of the absorption of carbon dioxide emitted in the decarbonation of the raw materials utilized in the manufacture of Portland cement clinker has taken place in 50 years.
- Due to the high porosity of the mortar, it carbonates at a higher rate, which is why it undergoes special treatment [32].

The carbon dioxide uptake estimation has been performed by means of the simplified method in which Equations (3) and (4) were employed. The values for α and β were 0.20 (service life) and 0.03 (end-of-life and secondary use), respectively.

ACDU (service life) = α × IPCC reported emissions due to the calcination process(3)

ACDU (end-of-life) = β × IPCC reported emissions due to the calcination process(4)

This method provides an easy and reliable calculation for estimating the annual carbon dioxide uptake (ACDU) in cement-based materials during its use stage, end-of-life stage, and secondary use, based on the reported calcination carbon dioxide emissions [32].

## 3. Results and Discussion

**Figure 3:**
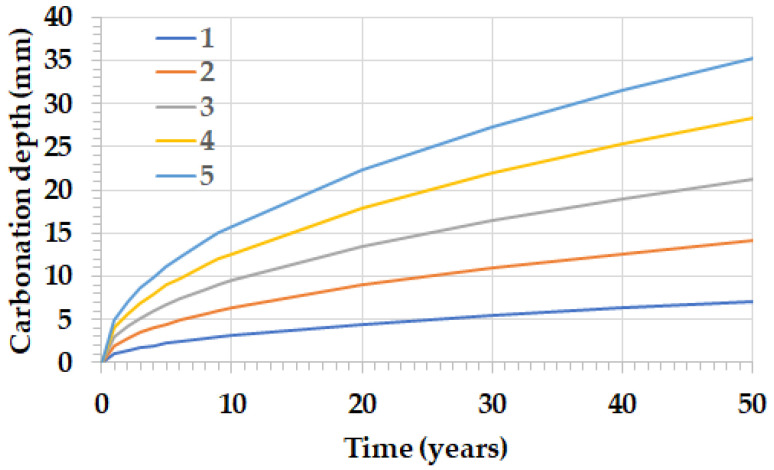
CO_2_ uptake according to the square root of time rule considering five carbonation coefficients from 1 to 5 mm/year^0.5^.

The carbon dioxide emitted during the calcination process of the raw materials in the production of Portland cement clinker is partially reabsorbed over the service life and end-of-life of the concrete structures and mortars made with cements manufactured with such clinker. The absorption of carbon dioxide follows, in a simplified form, the square law of time, as shown in Figure 3. It can be seen that the rate of carbonation depends on the carbonation coefficient, which depends on the quality (compactness, cement content, etc.) of the concrete or mortar. In the present work, an average value of the carbonation coefficient of 3 mm/year^0.5^ has been considered. In this way, the amount of carbon dioxide that has been absorbed each year has been calculated.

It was also assumed that 23% of the carbon dioxide emitted by the calcination stage in the manufacture of Portland cement clinker is completed after 50 years of exposure.

**Figure 4:**
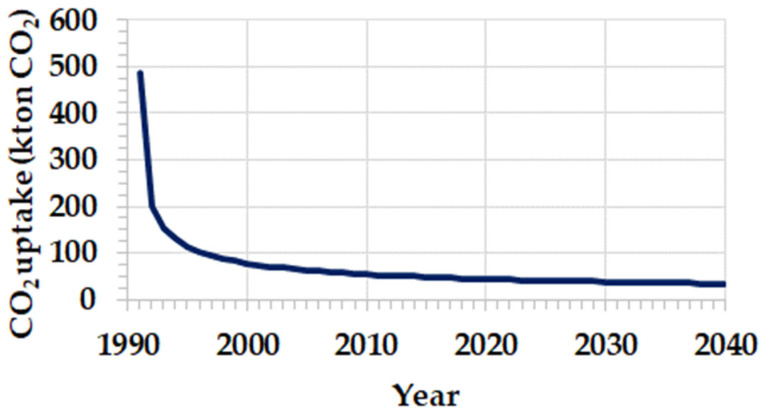
Carbon dioxide absorption by mortar and concrete made from Portland cement clinker produced in 1990. In that year, 14,904 ktons of carbon dioxide were emitted due to the calcination process, 3429 ktons of which are currently being absorbed over a period of 50 years.

For example, in the production of clinker in 1990, 14,904 ktons of carbon dioxide were emitted due to the calcination process, of which 3429 ktons of carbon dioxide will be absorbed over the next 50 years (Figure 4). It can be seen that the highest absorption of carbon dioxide occurs during the first years of the service life of the concrete structure or the setting of the mortar. In the example shown in Figure 4, it can be observed that, since the 20th year onwards, the carbon dioxide absorption follows an almost asymptotic trend.

Therefore, in order to account for the yearly carbon dioxide uptake, the partial carbonation occurring in each year under consideration of mortar and concrete produced over the fifty preceding years must be added up. Such CO_2_ uptake is distributed over the 50 years following their production and use according to the ‘square root of time’ formula, as shown in Figure 3.

**Figure 5:**
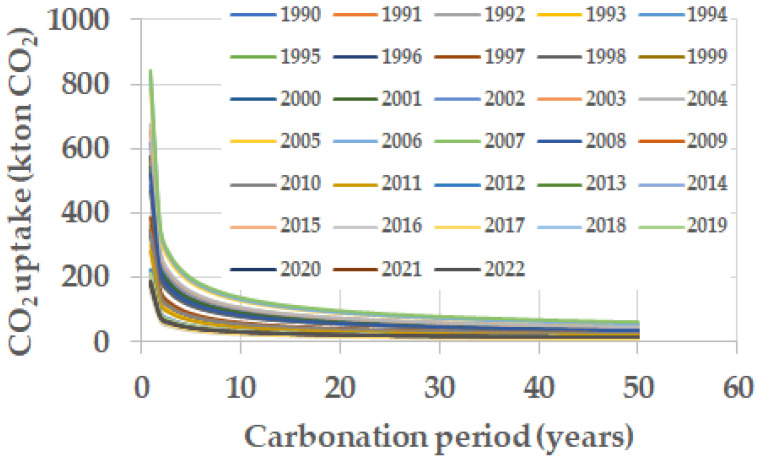
Carbon dioxide absorption by mortar and concrete made from Portland cement clinker produced from 1990 to 2022. In that period of time, 393,895 ktons of carbon dioxide were emitted due to the calcination process, 93,556 ktons of which are currently being absorbed over a period of 50 years.

Figure 5 shows the carbon dioxide absorption by mortar and concrete made from Portland cement clinker produced from 1990 to 2022. Globally, 400 megatons of carbon dioxide were emitted, 90 megatons of which are expected to be reabsorbed. The Intergovernmental Panel on Climate Change (IPCC) welcomes, in the Working Group I contribution to the Sixth Assessment Report (AR6), the natural carbonation process as a way of carbon offsetting by mortar and concrete [21].

Furthermore, according to EN 15978 [25], carbon dioxide uptake should be considered in B1 and C3 stages (Figure 1). Then, buildings can play an important role as carbon sinks [22] since they can permanently absorb carbon dioxide from the atmosphere. What is needed, then, is an adequate policy framework to enhance the development of new breakthrough technologies that would allow the cement sector a significant reduction in carbon dioxide emissions [13]. More specifically, the cement industry can offer carbon removal down the cement and concrete value chain in mortar and concrete through their service life by natural carbonation and/or during concrete production (ready-mix concrete or precast concrete) by means of controlled accelerated concrete carbonation. Both physicochemical processes permanently store carbon dioxide and increase the concrete’s compressive strength.

**Figure 6:**
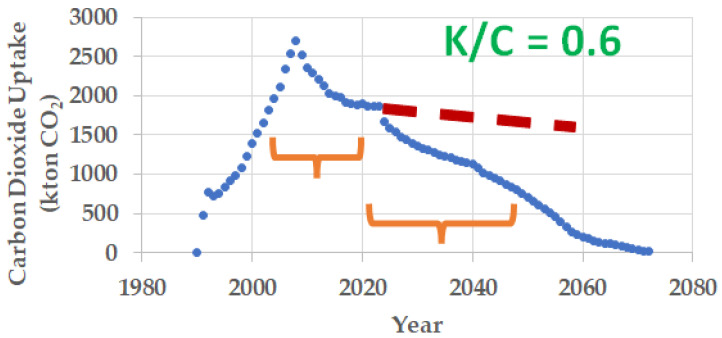
Carbon dioxide absorption by mortar and concrete made from Portland cement clinker produced from 1990 to 2022 in Spain over the 50 years following their production.

Figure 6 shows the carbon dioxide absorption by mortar and concrete made from Portland cement clinker produced from 1990 to 2022 in Spain over the 50 years following production.

The decrease in carbon dioxide uptake from 2007 to 2022 is due to the sharp reduction of cement production and consumption in Spain in that period of time. For 2022 to 2050, only the cement production from 1990 to 2022 was taken into account since the actual data of clinker production from 2023 to 2050 is unknown. Accordingly, the absorption results in that period of time are underestimated. In order to avoid this fact, a clinker production forecast from 2023 to 2050 has been made (dashed line in red) in order to provide a more complete scenario in Figure 6. It has been taken into account that on the one hand there is a general forecast of an increase in the production of Portland cement, but also that these cements will present a lower clinker factor (clinker-to-cement ratio in the Portland cement) in accordance with the Spanish cement sector roadmap, i.e., from the year 2023 to 2050, there will be a decrease in the clinker factor from 80% in 2022 to 75% in 2030, 70% in 2040, and 65% in 2050 [16,17].

Scientific assessments gathered by the Intergovernmental Panel on Climate Change (IPCC) to inform policymakers on the state of scientific evidence related to the mitigation of climate change, such as that regarding carbon dioxide uptake by construction materials, include the related uncertainties. However, the scientific complexity of carbon dioxide uptake estimation, among other climate change issues, presents a major challenge to academics in this field. Then, the IPCC has created a common uncertainty language that researchers from different disciplines can use to describe scientific evidence and related uncertainties [49]. Thus, the IPCC Fifth Assessment Report on Consistent Treatment of Uncertainties (henceforth: IPCC guidance note) [49] carefully reviews how to use confidence levels to outline the quality of evidence and the likelihood terms, considering that the findings from IPCC reports normally inform policy decisions on climate change mitigation. This IPCC guidance note [49] encourages researchers to assign confidence levels to the evidence by using the terms “very low”, “low”, “medium”, “high”, and “very high”. Utilization of confidence levels has raised since the first assessment report published by the Intergovernmental Panel on Climate Change (IPCC) in 1990 [50].

Estimation of carbon dioxide uptake by cement-based materials involves interdisciplinary efforts to assess scientific evidence for policymakers. Accordingly, the results presented in this study are classified as “very high confidence” since these findings are robust; they are based on consistent lines of high-quality research [48] and there is a great scientific agreement on concrete carbonation estimation, which is well-established in the scientific community [32,51]. In addition, the likelihood term associated with the projection of carbon dioxide uptake presented in this study is “very likely”, referring to the 90–100% probability according to the IPCC guidance note which translates each likelihood term into a probability interval [49]. To sum up, the uncertainty is presented in a manner that relates to decision-makers’ concerns [52].

**Figure 7:**
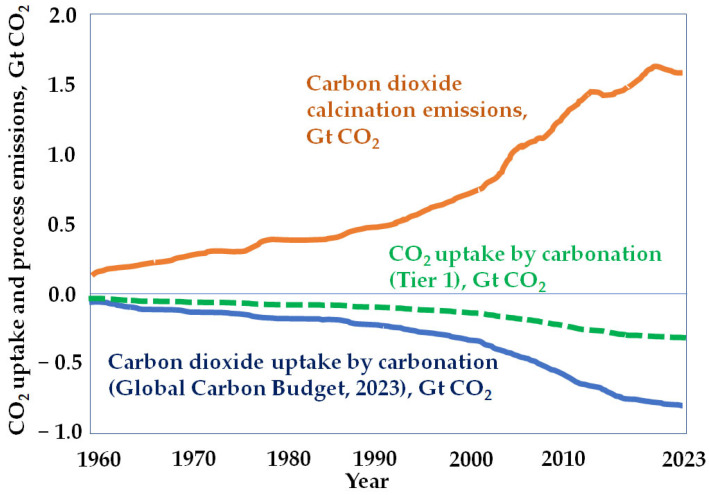
Worldwide carbon dioxide uptake by mortars and concretes from 1960 to 2023 estimated by the Global Carbon Budget report compared with the ones estimated by applying Tier 1 «source: author’s original».

The results obtained by applying Tier 1 are more conservative than those presented in the recent Global Carbon Budget report [53] published on 5 December 2023 (Figure 7). This document reported above 700 Mtons/year in 2023 for the cement carbonation sink. This value is like the one reported by the IPCC (about 50% of process emissions) [22].

Recently, the 28th Conference of the Parties of the UNFCCC (COP 28) in Dubai, UAE, recognized the need for deep, rapid, and sustained reductions to limit temperature rises to 1.5 °C above pre-industrial levels. However, decarbonizing the cement industry will take the collective effort of the stakeholders all working together, i.e., governments, civil society, policymakers, finance, scientists, and industry. Natural carbonation, known as (re-)carbonation, and controlled accelerated concrete carbonation can be considered as key initiatives that will help enable the shift to net-zero carbon emissions. Both processes permanently store carbon dioxide. In addition, it should be highlighted that the cement sector provides an essential material throughout the world, which enables sustainable and resilient infrastructure and housing to meet the present needs of society.

Reducing carbon dioxide emissions remains the primary, preferred, and most effective response to climate change. However, significantly reducing emissions will require a whole range of policies and measures and could be insufficient to lower the residual “hard to abate” carbon dioxide emissions. Therefore, carbon dioxide removal (CDR) can play a key role in the offsetting of emissions.

The Intergovernmental Panel on Climate Change (IPCC) has made it clear that carbon dioxide removal (CDR) is a critical tool to reach net zero emissions by 2050 [22] in order to neutralize residual carbon dioxide emissions once all mitigation efforts have been exhausted. Furthermore, according to the Article 4.1 of the Paris Agreement, a “balance between anthropogenic emissions by sources and removals by sinks of greenhouse gases” is expected to be achieved “in the second half of this century”. Meeting the Paris temperature goal of 1.5 °C calls for accelerating carbon dioxide emission reductions, increasing conventional carbon dioxide removals (CDR), and scaling up novel carbon dioxide removal (CDR), since an additional 0.8 to 2.9 gigatons of carbon dioxide per year of removal capacity will be necessary by 2030 [54]. Thus, all the carbon dioxide removal, including natural concrete carbonation and controlled accelerated concrete carbonation, should be considered as soon as possible to ensure 2030 scenarios are achievable.

Given carbon dioxide removal’s relevance to achieving net-zero commitments, the cement industry has a detailed net zero carbon emissions commitment and pathway, including cement-based material carbonation. In addition, for companies applying for the net-zero certification under the Science-Based Targets initiative’s (SBTi’s) Corporate Net-Zero Standard, after they have depleted decarbonization actions, they have to neutralize any residual emissions [55].

The cement industry offers carbon dioxide removal solutions down the cement and concrete value chain comprising both nature-based removal (natural carbonation) and technology-based removal (controlled accelerated concrete carbonation and Carbon Capture, Utilization, and Storage (CCUS)). In particular, nature-based removal, such as natural carbonation, take away carbon dioxide by restoring ecosystems; can play a key role in removals over the long-term; and are also a more cost-effective path [56].

Both natural carbonation and controlled accelerated concrete carbonation deliver “durable” removal by storing carbon dioxide permanently with minimal risk of release into the atmosphere. These durable solutions are preferable to ensure removal measures remain effective in the long term. Rather, this paper assumes that the carbon dioxide uptake by natural carbonation is 23% of the carbon dioxide process emissions over the lifetime (20%) and end-of-life (3%) of mortar and concrete structures.

## 4. Conclusions

The uptake of carbon dioxide by Spanish cement-based materials produced from 1990 to 2020 has been estimated by using a simplified method named Tier 1. This method considers the carbon dioxide released by the calcination process developed during the clinker production, i.e., process emissions. The chief conclusions of this study can be summarized as follows:

Ninety megatons of carbon dioxide has been estimated as the uptake by cement-based materials made with Portland cement clinker produced in Spain from 1990 to 2022.

The simplified method known as Tier 1 used in the present study is a conservative estimation method to consider the carbon dioxide uptake by cement-based materials that provides relevant information to fill the gap between emissions and absorption (mitigation) in the climatic models. Furthermore, this feasible method can be applied in anywhere. Therefore, this easy method should be implemented by the IPCC in the next refinement to the 2006 and 2019 IPCC guidelines for national greenhouse gas inventories.

Finally, natural carbonation can play a key role in removal over the long-term and is also a more cost-effective path than other nature-based removals. And, in this sense, it is worth highlighting that we propose the use of blended cements in mortar and concrete as a way to increase carbon dioxide uptake in cement-based materials.

## Data Availability

Data are contained within the article.
